# Drug-Coated Balloon-Oriented Angioplasty for Severe Symptomatic Atherosclerotic MCA Stenosis in Young Adults

**DOI:** 10.3389/fneur.2021.743851

**Published:** 2022-02-01

**Authors:** Yuyan Zhang, Xi Chu, Yao Meng, Jun Zhang, Lili Sun, Wei Zhao, Meimei Zheng, Hao Yin, Wei Wang, Jinping Zhang, Yun Song, Ju Han

**Affiliations:** Department of Neurology, Shandong Provincial Qianfoshan Hospital, The First Affiliated Hospital of Shandong First Medical University, Jinan, China

**Keywords:** drug-coated balloon, young patients, angioplasty, middle cerebral artery (MCA), stenosis

## Abstract

**Purpose:**

The clinical significance of endovascular therapy in young patients with symptomatic intracranial atherosclerotic stenosis is not clear. We aimed to report our preliminary findings on the safety and effectiveness of a new endovascular treatment strategy, drug-coated balloon (DCB)-oriented angioplasty for young adults with severe symptomatic atherosclerotic middle cerebral artery (MCA) stenosis.

**Methods:**

We retrospectively retrieved the data of seven young patients aged 21–32 years old with severe symptomatic atherosclerotic MCA stenosis treated with DCB-oriented angioplasty from January 2018 to October 2020. We collected clinical presentations, perioperative complications, and clinical and radiological outcomes.

**Results:**

The DCB was successfully dilated at the lesions in all seven patients and good antegrade perfusion was achieved in five. The other two patients underwent remedial stent implantation due to residual stenosis >50% and unstable antegrade perfusion after DCB dilatation. However, none of the patients had perioperative complications. There were no recurrent ischemic symptoms after a mean clinical follow-up period of 15.4 ± 6.9 months. Repeat vascular images of the patients showed no restenosis at 3- or 6-month imaging follow-up periods. High-resolution MRI (HRMRI) was completed in all the patients before the angioplasty procedure and at follow-up. Focal eccentric wall thickening was present at the site of stenosis preoperatively; however, the plaques had disappeared by the time of follow-up for all patients.

**Conclusion:**

DCB-oriented angioplasty may be safe and effective for young patients with severe symptomatic atherosclerotic MCA stenosis.

## Introduction

Approximately 10–15% of ischemic strokes occur in young adults (1). Stroke due to early-onset atherosclerosis accounts for 20–25% (2), which is even higher in China. Middle cerebral artery (MCA) stenosis accounts for a high proportion of young patients with symptomatic intracranial atherosclerotic stenosis (ICAS) (3). Even after aggressive medical treatment according to the Stenting and Aggressive Medical Management for Preventing Recurrent Stroke in Intracranial Stenosis (SAMMPRIS) trial (4), almost 20% of patients had a recurrent stroke in the real world (5). Recent studies have shown that drug-coated balloons (DCB) are effective in preventing in-stent restenosis (ISR) (6), and preliminary data suggest that they are superior to stent implantation in the treatment of patients with symptomatic ICAS (7). This new endovascular treatment strategy, DCB-oriented angioplasty, might become an alternative treatment for intracranial atherosclerotic diseases (8). So far, however, there is no relevant report on DCB-oriented angioplasty in young patients with ICAS. Therefore, our study intends to explore the short-term clinical and imaging outcomes of DCB angioplasty in young patients with severe MCA stenosis.

## Methods

### Study Population

The data that support this study's findings are available upon reasonable request. Based on our prospectively maintained stroke database, we retrospectively retrieved the data of young stroke patients with severe symptomatic atherosclerotic MCA stenosis (≥70% degree of stenosis) between January 2018 and October 2020. All patients gave their informed consent, and the institutional review board approved the study.

The inclusion criteria were as follows:

1. Patients ranged from 18 to 35 years old.
2. MCA stenosis confirmed by digital subtraction angiography (DSA).
3. Atherosclerotic stroke was confirmed based on the following evidence: (a) presence of vascular atherosclerotic risk factors (e.g., hypertension, diabetes, dyslipidemia, smoking, family history of atherosclerotic diseases, or peripheral atherosclerosis) and (b) eccentric wall thickening on high-resolution MRI (HRMRI).
4. Patients suffered from recurrent transient ischemic attack (TIA), ischemic stroke, or progressive stroke in the MCA territory after treatment with dual antiplatelet therapy (DAPT) in combination with rigid control for hypertension, hyperlipidemia, and diabetes mellitus.
5. Arterial spin labeling (ASL) assessed a large area of low perfusion of MCA territory.

The exclusion criteria were as follows:

1. Non-atherosclerotic disease (suspected cerebral vasculitis, arterial dissection, moyamoya disease, and potential cardioembolism).
2. Stroke caused by perforator artery occlusion or embolism.
3. Large infarction core shown on diffusion-weighted imaging (DWI).

### Procedures

An experienced interventional neuroradiologist performed all procedures using general anesthesia. The angiographic machine in our center was mono-plane. Stenosis grades were determined according to the Warfarin-Aspirin Symptomatic Intracranial Disease (WASID) study (5). All lesions were initially predilated with a conventional balloon (Gateway balloon, Boston Scientific, USA) with a balloon to vessel ratio of 0.8–1.0, which was supposed to facilitate the advancement of the DCB and drug penetration. Then DCB (SeQuent Please, B. Braun, Germany) was delivered to the lesions (9, 10). The conventional balloons and DCBs were left inflated for 60 s with 6 atm. All patients, or their authorized family members, provided informed consent for off-label use of coronary DCB. After the withdrawal of the DCB, angiography was performed. The intervention ended with a residual stenosis ≤50% and stable antegrade perfusion, without vascular dissection, perforation, or distal embolization. If the residual stenosis was >50%, or if there was a vessel dissection after DCB dilation, remedial self-expanding stenting (Wingspan stent, Stryker Neurovascular, USA) implantation was performed. The details of the DCB-oriented angioplasty protocol were as previously described (11).

All patients were routinely on DAPT (100 mg/day aspirin and 75 mg/day clopidogrel) for at least 5 days prior to the intervention. Thromboelastography platelet mapping was performed to guide the regulation of antiplatelet therapy. Intravenous heparin boluses were administered to maintain the activated clotting time between 250 and 300 s during the procedure. Immediately after the procedure, brain CT was performed, and the patients were sent to the neurological intensive care unit for continuous observation and blood pressure monitoring, with strict control of blood pressure. Three or 6 months after the intervention, DAPT was changed to mono ATP, based on the intervention strategy. Specifically, patients changed to mono ATP after 3 months if with DCB dilation only, and after 6 months if with DCB dilation plus stenting implantation. In addition, they were prescribed statins and received education to control for other risk factors.

### HRMRI Neuroimaging Protocol

All patients were imaged by a 3.0-T MR scanner (GE, Discovery MR 750) with a standard 32-channel head coil. The imaging protocol included a plane and an enhanced 3D CUBE T1W HRMRI. The 3D CUBE T1W HRMRI image parameters were as follows: repetition time (TR)−600 ms; echo time (TE)−13.5 ms; slice thickness−1 mm; acquisition matrix−288 × 288; and scan plane—sagittal. The fat suppression technique was used to reduce fat signals from the scalp. The resulting voxel size was 0.4 × 0.4 × 0.4 mm, and 339 coronal images covering the whole brain were obtained with the scan time of 4 min and 16 s. After the 3D CUBE T1W HRMRI scan, an enhanced 3D CUBE T1W HRMRI scan was completed 2 min after the intravenous injection of Gd-DTPA (0.1 mmol/kg) contrast agent.

### Statistical Analysis

Quantitative data were expressed as means ± SD or as medians with interquartile range (IQR), whereas categorical data were presented as numbers and percentages. All statistical analyses were performed using SPSS 22 software (SPSS Inc., Chicago, United States).

## Results

**Figure 1:**
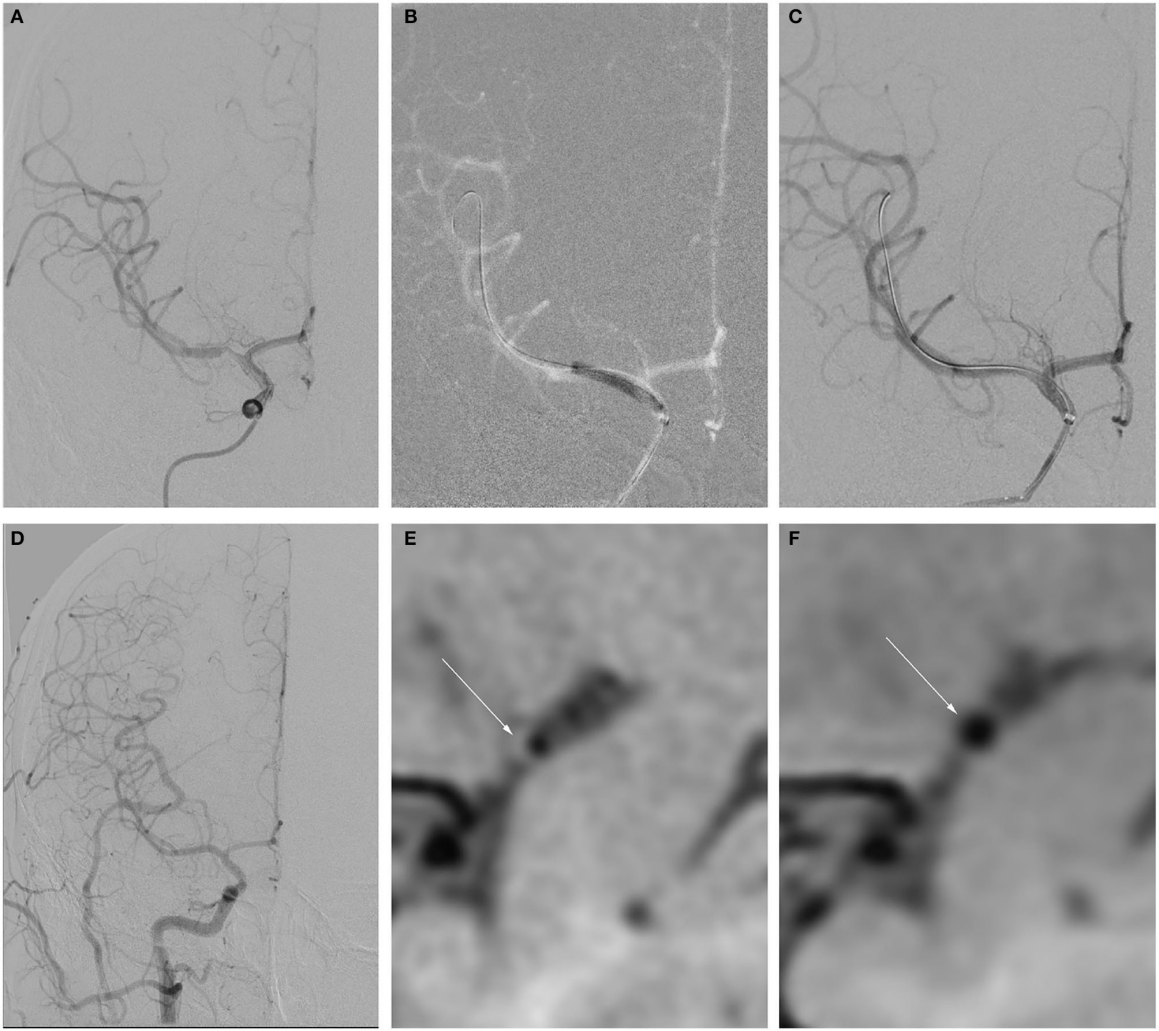
Example of drug-coated balloon (DCB) dilation and follow-up. **(A–F)** Angiography and high-resolution MRI (HRMRI) results of the DCB dilatation during the procedure and follow-up. **(A)** Severe right middle cerebral artery (MCA) stenosis. **(B)** DCB dilatation after predilatation. **(C)** The angiographic result after the procedure. **(D)** Angiographic result at 3.3-month follow-up. **(E)** Focal eccentric plaque identified at target vessel wall [arrow in **(E)**] in high-resolution T1-weighted imaging sequence. **(F)** No plaque or wall thickening was identified on high-resolution T1-weighted imaging sequences at 3.3-month follow-up.

**Figure 2:**
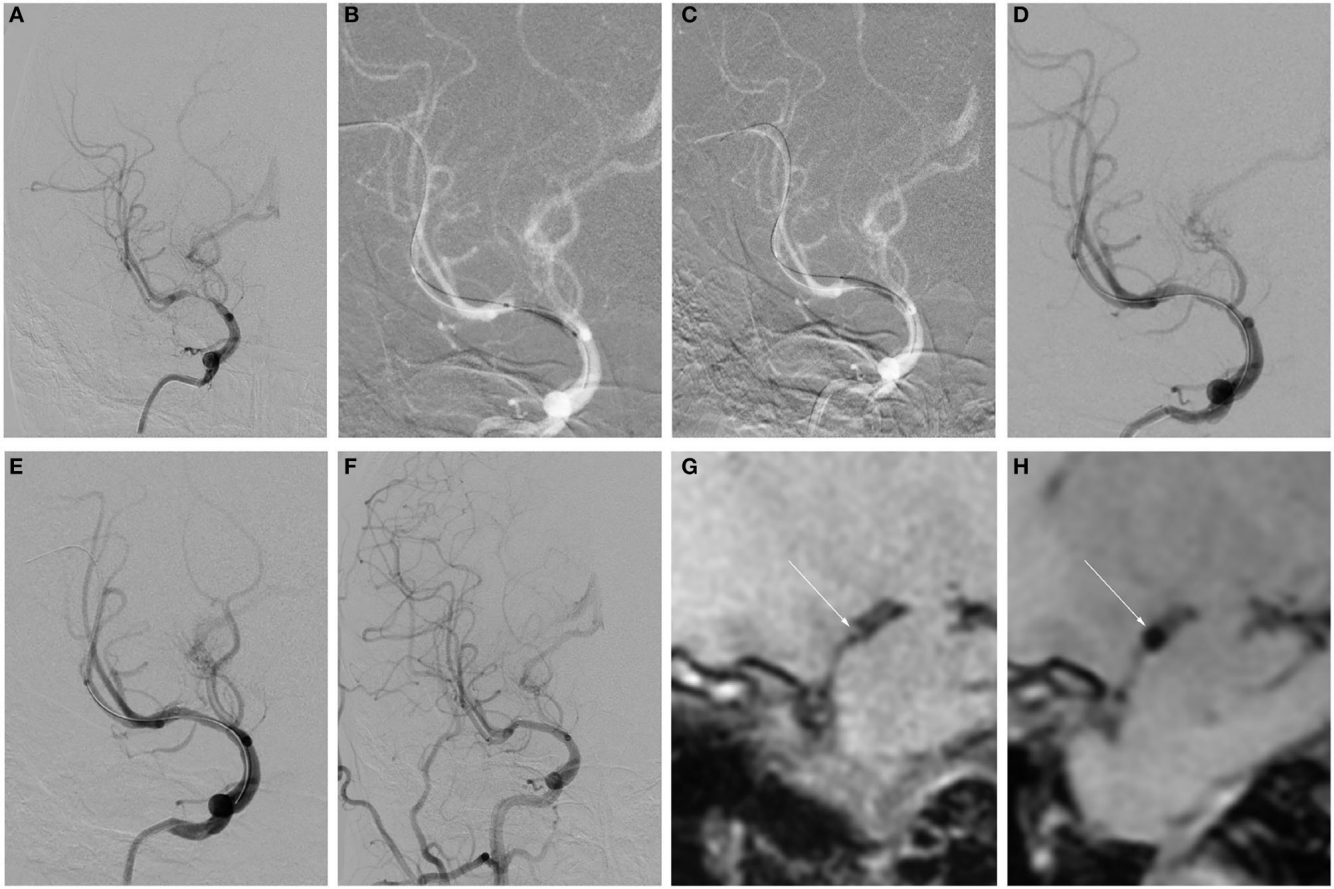
Example of remedial stent implantation after DCB dilatation and follow-up. **(A–H)** Angiography and HRMRI results of remedial stent implantation after DCB dilatation during the procedure and follow-up. **(A)** Severe right MCA stenosis. **(B)** Predilatation with a conventional balloon. **(C)** DCB dilatation after predilatation. **(D)** The angiographic result after DCB. **(E)** The angiographic result after remedial stent implantation. **(F)** Angiographic result at 5.5-month follow-up. **(G)** Focal eccentric plaque identified at target vessel wall [arrow in **(G)**] in high-resolution T1-weighted imaging sequence. **(H)** Slight circumferential wall thickening was identified on a high-resolution T1-weighted imaging sequence at a 5.5-month follow-up.

Seven patients were enrolled in this study; Table 1 shows their baseline demographic and clinical characteristics. The mean age was 28.4 ± 3.9 years. The median time from initial radiological diagnosis to endovascular treatment was 21 days (IQR, 16–33), and the median time from the onset of the last symptom to endovascular treatment was 15 days (IQR, 2–20). The procedures and imaging characteristics and clinical outcomes of the patients are detailed in Tables 2, 3. Five patients were treated with DCB only (Figure 1), whereas two were treated with remedial stenting implantation due to residual stenosis >50% after DCB dilatation (Figure 2). This might be due to elastic recoil of the artery, and successful recanalization was achieved in all patients. The median residual stenosis rate was 0% (IQR, 0–20%). None of the patients had perioperative complications, and they all completed their clinical and imaging follow-up. During the mean clinical follow-up period of 15.4 ± 6.9 months, none of the patients experienced ischemic stroke. DSA was performed in four patients and CTA in one, and two patients were assessed *via* MRA during the vessel imaging examination with a mean follow-up time of 6.6 ± 4.2 months (Figures 1, 2). The median stenosis rate at follow-up was 0% (IQR, 0–0%). None of the patients had restenosis at the lesion site, and residual stenosis was reduced in two of them compared with their immediate postoperative DSA. The residual stenosis was reduced from 50% postoperatively to 20% at the 3-month follow-up in Patient 2, and from 20 to 0% at the 1-year follow-up in Patient 4. HRMRI was conducted in all patients before the procedure and at the follow-up to observe vessel wall and plaque changes before and after the interventional therapy. Before the procedure, all the patients showed focal eccentric plaques at the lesion site. Four patients showed mild to moderate plaque enhancement on contrast enhancement, whereas three showed significant enhancement, which reflected plaque instability. However, at follow-up, they all showed the disappearance of plaque at the lesion site, and three patients had mild circumferential enhancement of the arterial wall (Figures 1, 2).

**Table 1:** Baseline clinical characteristics.

**Patient number**	**Age**	**Sex**	**Risk factor**	**Symptoms**	**Time from last onset to treatment (days)**	**Time from image stenosis to treatment (days)**
1	27	M	Smoking, stroke, hypertension	Recurrent limb weakness, slurred speech	20	21
2	31	M	Smoking	Recurrent limb weakness and numbness	24	23
3	32	M	Smoking	Recurrent slurred speech, limb shaking	13	19
4	29	M	Hypertension,diabetes mellitus	Recurrent limb weakness, slurred speech	15	404
5	27	M	Smoking, hypertension, hyperlipidemia	Recurrent slurred speech, limb numbness, dizziness	17	16
6	32	M	Smoking, stroke	Recurrent limb weakness and numbness	2	33
7	21	M	Smoking	Recurrent limb weakness and shaking, facial numbness	1	16

**Table 2:** Angiographic and procedural characteristics.

**Patiet number**	**Stenosis length (mm)**	**Stenosis degree (%)**	**Mori classification**	**CBA**	**DCBA**	**Stent**
1	11	99	B	1.5 × 15 mm	2.5 × 15 mm	3.5 × 20 mm
				2.25 × 15 mm		
2	7	80	B	2.5 × 15 mm	2.5 × 20 mm	N
3	10	95	B	2.0 × 15 mm	2.0 × 17 mm	N
4	9	99	B	2.25 × 15 mm	2.5 × 20 mm	N
5	5	90	A	2.5 × 15 mm	2.5 × 17 mm	N
6	12	90	B	2.5 × 15 mm	2.5 × 17 mm	3.0 × 15 mm
7	6	80	B	2.5 × 15 mm	2.75 × 17 mm	N

*CBA, conventional balloon angioplasty; DCBA, drug-coated balloon angioplasty; Y, yes; N, no*.

**Table 3:** Clinical and imaging outcomes in-hospital and during follow-up.

**Patiet number**	**Immediate postoperative residual stenosis (%)**	**Perioperative complications**	**Stenosis degree at follow-up (%)**	**Recurrent symptoms**
1	0	N	0	N
2	50	N	20	N
3	0	N	0	N
4	20	N	0	N
5	0	N	0	N
6	0	N	0	N
7	0	N	0	N

*Y, yes; N, no*.

## Discussion

In China, the proportion of patients with early-onset atherosclerotic stroke is increasing. Young patients lose as much as 20% of their potential life years due to stroke, and they have a high life expectancy; therefore, avoiding major deficits in young patients has the potential to obtain significant social benefits. Although endovascular treatment has been increasingly recognized and recommended, to our knowledge, no report has focused on interventional therapy in young patients with intracranial atherosclerotic stenosis.

Since the negative results of the SAMMPRIS (4) and VISSIT (12) trials, medical therapy has been considered as the standard treatment for symptomatic ICAS. However, the Groupe d'Etude des Stenoses Intra-Craniennes Atheromateuses symptomatiques (GESICA) Study (13) demonstrated that 38.2% of patients with symptomatic intracranial stenosis experienced a recurrent cerebrovascular event in the territory of the stenotic artery at their 2-year follow-up despite optimal medical treatment. For these patients, aggressive medical treatment alone was not enough to prevent secondary stroke. The study by Gao et al. (14) and the WEAVE trial (15) revealed that improvements in patient selection and procedures can greatly reduce periprocedural complications and events after intracranial stenting implantation. Moreover, the WOVEN trial (16) found relatively low 1-year stroke and death rates in stented patients, and there appeared to be a clinical benefit of stenting at the 1-year follow-up. DCB can inhibit intimal hyperplasia. It can effectively reduce the occurrence of vascular restenosis in intracranial interventional therapy while maintaining the overall safety of the procedure and avoiding permanent implants in some patients (8, 11, 17, 18). Based on these findings, we speculated that the new endovascular treatment strategy (DCB-oriented angioplasty) could improve the long-term restenosis rate and thus benefit such patients.

What was encouraging to us was the fact that all patients in this study achieved good procedural and follow-up outcomes. Good antegrade perfusion was achieved in five of the seven patients with the application of the DCB alone. Only two patients received remedial stent implantation due to residual stenosis >50% and unstable antegrade perfusion after DCB dilatation. Because DCB has no residual metal mesh and polymer matrix, it can theoretically significantly reduce the intimal inflammatory response, shorten vascular endothelial healing time, reduce the risk of thrombosis, shorten DAPT time, and reduce the risk of bleeding. At the same time, DCB prevents foreign body placement and also preserves the opportunity for subsequent treatment when necessary for the patient (19). This new endovascular treatment strategy is particularly suitable for young people and drives young patients to be somewhat more aggressive in treatment. In our study, none of the patients had restenosis at the lesion site at follow-up. Of note, residual stenosis was relieved at follow-up compared to the immediate postoperative DSA in two patients. Inhibition of plaque progression or vascular remodeling associated with local paclitaxel delivery is a possible mechanism. Experimental animal studies have demonstrated that paclitaxel causes apoptosis and necrosis of endothelial and smooth muscle cells (20). This suggests that the use of coated paclitaxel could prevent intimal hyperplasia and arterial constriction (21).

HRMRI for intracranial arteries is an important technique for evaluating the vessel wall and plaque of the intracranial artery *in vivo*, and it provides good consistency. All patients in this study showed disappearance of plaque at the lesion site on HRMRI at follow-up, and we speculated that this may be because DCB squeezes the plaque when it expands, causing the plaque to redistribute and migrate into the wall, which organizes and absorbs it. Three patients had good lumen patency on follow-up DSA and mild circumferential wall enhancement on HRMRI. Long-term follow-up and statins may be required for such patients. The mechanism of this imaging feature has not been clear, and further pathology is still required for confirmation. HRMRI is non-invasive and is more acceptable to patients at follow-up. It can not only observe the degree of stenosis but also see the evolution of plaque, which helps us comprehensively observe the target vessel lumen and wall.

Our study has several limitations. First, this is a retrospective study that may have selection bias. Second, the number of patients was limited, and the results should be extrapolated with caution.

## Conclusions

The choice of treatment strategy is still a dilemma for young patients with severe symptomatic atherosclerotic MCA stenosis. Our preliminary results showed that DCB-oriented angioplasty was safe with complete reperfusion and good functional outcome. Our case series highlights a possible superior treatment modality for young symptomatic ICAS patients that seems to be a promising endovascular treatment strategy compared to conventional stenting angioplasty. Further studies with a larger sample size are needed to confirm our findings.

## Data Availability Statement

The original contributions presented in the study are included in the article/supplementary material, further inquiries can be directed to the corresponding author/s.

## Ethics Statement

The studies involving human participants were reviewed and approved by the Institutional Review Board of The First Affiliated Hospital of Shandong First Medical University. The patients/participants provided their written informed consent to participate in this study. Written informed consent was obtained from the individual(s) for the publication of any potentially identifiable images or data included in this article.

## Author Contributions

JH: study concept, design, and study supervision. YZ, XC, and YM: cases data collection. JH, JuZ, and YZ: analysis and interpretation of the data. JH and YZ: drafting of the manuscript. All authors were involved in critically analyzing the article, approved the contents of the article, and also agree to be accountable for all aspects of the work submitted for publication.

## Conflict of Interest

The authors declare that the research was conducted in the absence of any commercial or financial relationships that could be construed as a potential conflict of interest.

## Publisher's Note

All claims expressed in this article are solely those of the authors and do not necessarily represent those of their affiliated organizations, or those of the publisher, the editors and the reviewers. Any product that may be evaluated in this article, or claim that may be made by its manufacturer, is not guaranteed or endorsed by the publisher.

## References

[1] Hathidara MY Saini V Malik AM. Stroke in the young: a global update. Curr Neurol Neurosci Rep. (2019) 19:91. 10.1007/s11910-019-1004-1 31768660

[2] Varona JF Guerra JM Bermejo F Molina JA Gomez de la Cámara A. Causes of ischemic stroke in young adults, and evolution of the etiological diagnosis over the long term. Eur Neurol. (2007) 57:212–8. 10.1159/000099161 17268202

[3] von Sarnowski B Schminke U Tatlisumak T Putaala J Grittner U Kaps M. Prevalence of stenoses and occlusions of brain-supplying arteries in young stroke patients. Neurology. (2013) 80:1287–94. 10.1212/WNL.0b013e31828ab2ed 23468548

[4] Derdeyn CP Chimowitz MI Lynn MJ Fiorella D Turan TN Janis LS. Aggressive medical treatment with or without stenting in high-risk patients with intracranial artery stenosis (SAMMPRIS): the final results of a randomised trial. Lancet. (2014) 383:333–41. 10.1016/S0140-6736(13)62038-3 24168957 PMC3971471

[5] Chimowitz MI Lynn MJ Howlett-Smith H Stern BJ Hertzberg VS Frankel MR. Comparison of warfarin and aspirin for symptomatic intracranial arterial stenosis. N Engl J Med. (2005) 352:1305–16. 10.1056/NEJMoa043033 15800226

[6] Vajda Z Güthe T Perez MA Kurre W Schmid E Bäzner H. Prevention of intracranial in-stent restenoses: predilatation with a drug eluting balloon, followed by the deployment of a self-expanding stent. Cardiovasc Intervent Radiol. (2013) 36:346–52. 10.1007/s00270-012-0450-9 22869043 PMC3595472

[7] Gruber P Garcia-Esperon C Berberat J Kahles T Hlavica M Anon J. Neuro Elutax SV drug-eluting balloon versus Wingspan stent system in symptomatic intracranial high-grade stenosis: a single-center experience J Neurointerv Surg. (2018) 10:e32. 10.1136/neurintsurg-2017-013699 29627786

[8] Zhang J Zhang X Zhang J Song Y Zheng M Sun L. Drug-coated balloon dilation compared with conventional stenting angioplasty for intracranial atherosclerotic disease. Neurosurgery. (2020) 87:992–8. 10.1093/neuros/nyaa191 32445576

[9] Jeger RV Eccleshall S Wan Ahmad WA Ge J Poerner TC. Shin ES. Drug-coated balloons for coronary artery disease: third report of the international DCB consensus group. JACC Cardiovasc Interv. (2020) 13:1391–402. 10.1016/j.jcin.2020.02.043 32473887

[10] Kleber FX Mathey DG Rittger H Scheller B; German Drug-eluting Balloon Consensus Group. How to use the drug-eluting balloon: recommendations by the German consensus group. EuroIntervention. (2011) 7 Suppl K:K125–K128. 10.4244/EIJV7SKA21 22027722

[11] Han J Zhang J Zhang X Zhang J Song Y Zhao W. Drug-coated balloons for the treatment of symptomatic intracranial atherosclerosis: initial experience and follow-up outcome. J Neurointerv Surg. (2019) 11:569–73. 10.1136/neurintsurg-2018-014237 30337378

[12] Zaidat OO Fitzsimmons BF Woodward BK Wang Z Killer-Oberpfalzer M Wakhloo A. Effect of a balloon-expandable intracranial stent vs medical therapy on risk of stroke in patients with symptomatic intracranial stenosis: the VISSIT randomized clinical trial. JAMA. (2015) 313:1240–8. 10.1001/jama.2015.1693 25803346

[13] Mazighi M Tanasescu R Ducrocq X. Vicaut E Bracard S Houdart E. Prospective study of symptomatic atherothrombotic intracranial stenoses: the GESICA study. Neurology. (2006) 66:1187–91. 10.1212/01.wnl.0000208404.94585.b2 16636236

[14] Gao P Wang D Zhao Z Cai Y Li T Shi H. Multicenter prospective trial of stent placement in patients with symptomatic high-grade intracranial stenosis. AJNR Am J Neuroradiol. (2016) 37:1275–80. 10.3174/ajnr.A4698 26869472 PMC7960346

[15] Alexander MJ Zauner A Chaloupka JC Baxter B Callison RC Gupta R. WEAVE trial: final results in 152 on-label patients. Stroke. (2019) 50:889–94. 10.1161/STROKEAHA.118.023996 31125298

[16] Alexander MJ Zauner A Gupta R Alshekhlee A Fraser JF. Toth G. The WOVEN trial: wingspan one-year vascular events and neurologic outcomes. J Neurointerv Surg. (2021) 13:307–10. 10.1136/neurintsurg-2020-016208 32561658

[17] Wang AY Chang CH Chen CC Wu YM Lin CM Chen CT. Leave nothing behind: treatment of intracranial atherosclerotic disease with drug-coated balloon angioplasty. Clin Neuroradiol. (2020) 31:35–4. 10.1007/s00062-020-00935-w 32720067

[18] Remonda L Diepers M Berberat J Kahles T Anon J Nedeltchev K. Drug-coated balloon treatment in symptomatic intracranial high grade stenosis: a retrospective study of 33 patients. Clin Neuroradiol. (2020) 31: 15–9. 10.1007/s00062-020-00936-9 32691077

[19] Gruber P. Remonda L. Device profile of different paclitaxel-coated balloons: neuro Elutax SV, Elutax '3' Neuro and SeQuent Please NEO for the treatment of symptomatic intracranial high-grade stenosis: overview of their feasibility and safety. Exp Rev Med Devices. (2020) 17:87–92. 10.1080/17434440.2020.1719829 31962054

[20] Sheehy A Hsu S Bouchard A Lema P Savard C Guy LG. Comparative vascular responses three months after paclitaxel and everolimus-eluting stent implantation in streptozotocin-induced diabetic porcine coronary arteries. Cardiovasc Diabetol. (2012) 11:75. 10.1186/1475-2840-11-75 22716997 PMC3413520

[21] Ann SH Her AY Singh GB Okamura T Koo BK Shin ES. Serial morphological and functional assessment of the paclitaxel-coated balloon for de Novo lesions. Rev Esp Cardiol. (2016) 69:1026–32. 10.1016/j.rec.2016.03.026 27321644

